# Managing an effective system for retrieving IVC filters: outcomes of a prospective patient database, 2012–2023

**DOI:** 10.1186/s42155-025-00550-1

**Published:** 2025-04-28

**Authors:** Monica M. Matsumoto, Ann Cun, Corinne DeSanto, Anna Paycardo, S. William Stavropoulos, Scott O. Trerotola

**Affiliations:** Section of Interventional Radiology, Department of Radiology, Penn Medicine, 1 Silverstein, 3400 Spruce Street, Philadelphia, PA 19104 USA

**Keywords:** Inferior vena cava filter, Filter retrieval, Interventional radiology, Patient database

## Abstract

**Purpose:**

To evaluate retrievable inferior vena cava (IVC) filter outcomes with a prospectively maintained database and active patient management by interventional radiology (IR).

**Materials & methods:**

Patients with retrievable IVC filters placed by IR from 2012 to 2023 at a single, tertiary institution were tracked in a prospective registry, and follow-up was organized by a designated IR physician assistant. Patients were contacted after the filter was placed by IR and a clinic visit arranged; filter removal was scheduled when deemed appropriate. Retrospective review of filter outcomes, including retrieval, patient death, and need for permanent filtration, was performed.

**Results:**

Over the 12-year study period, 607 retrievable IVC filters were placed: 516 Denali, 63 Eclipse, 19 Günther Tulip, and 9 Celect Platinum. In total, 43% (260) were retrieved, 12% (75) were adjudicated to be permanent, and 42% (253) died with the filter in place. The remaining 3% (19) comprised patients alive with the filter not yet retrieved at study endpoint, 42% (8/19) of which were placed in 2023. Of this cohort, 8 still needed the filter and were being monitored to determine follow-up timing, 2 needed a follow-up appointment, and 9 were lost to follow-up due to repeated no-shows and/or inability to reach the patient despite multiple attempts. Overall, 1.5% (9/607) of all filters placed were not accounted for.

**Conclusion:**

This study demonstrates high accountability (98.5%) of retrievable IVC filters when using a prospective registry actively managed by an IR PA, providing an effective and feasible model for facilitating appropriate follow-up.

## Introduction

Retrievable inferior vena cava (IVC) filters have been in use since 2003 when they were approved by the U.S. Food and Drug Administration (FDA) [1, 2]. They are used most commonly in patients with acute venous thromboembolism who have a contraindication to anticoagulation or who cannot be adequately treated on anticoagulation alone [3]. While retrievable IVC filters can remain in place indefinitely, best practices, including initial 2010 and updated 2014 FDA safety communications, recommend timely removal when the device is no longer indicated [3–5].

Several initiatives have been proposed and implemented to increase the rates of filter retrieval, including dedicated IVC filter clinics, patient and physician education, prospective patient registries, and automated reminders and follow-up scheduling, noting a “structured follow-up program” is recommended by Society of Interventional Radiology guidelines [3, 6, 7]. Varying success has been reported with interventions targeting filter retrieval, with removal rates ranging from 31 to 95% [6]. This study aims to describe the management process and outcomes of a longitudinal filter tracking program established by an interventional radiology (IR) section at a single tertiary academic center.

## Materials & methods

**Fig. 1:**
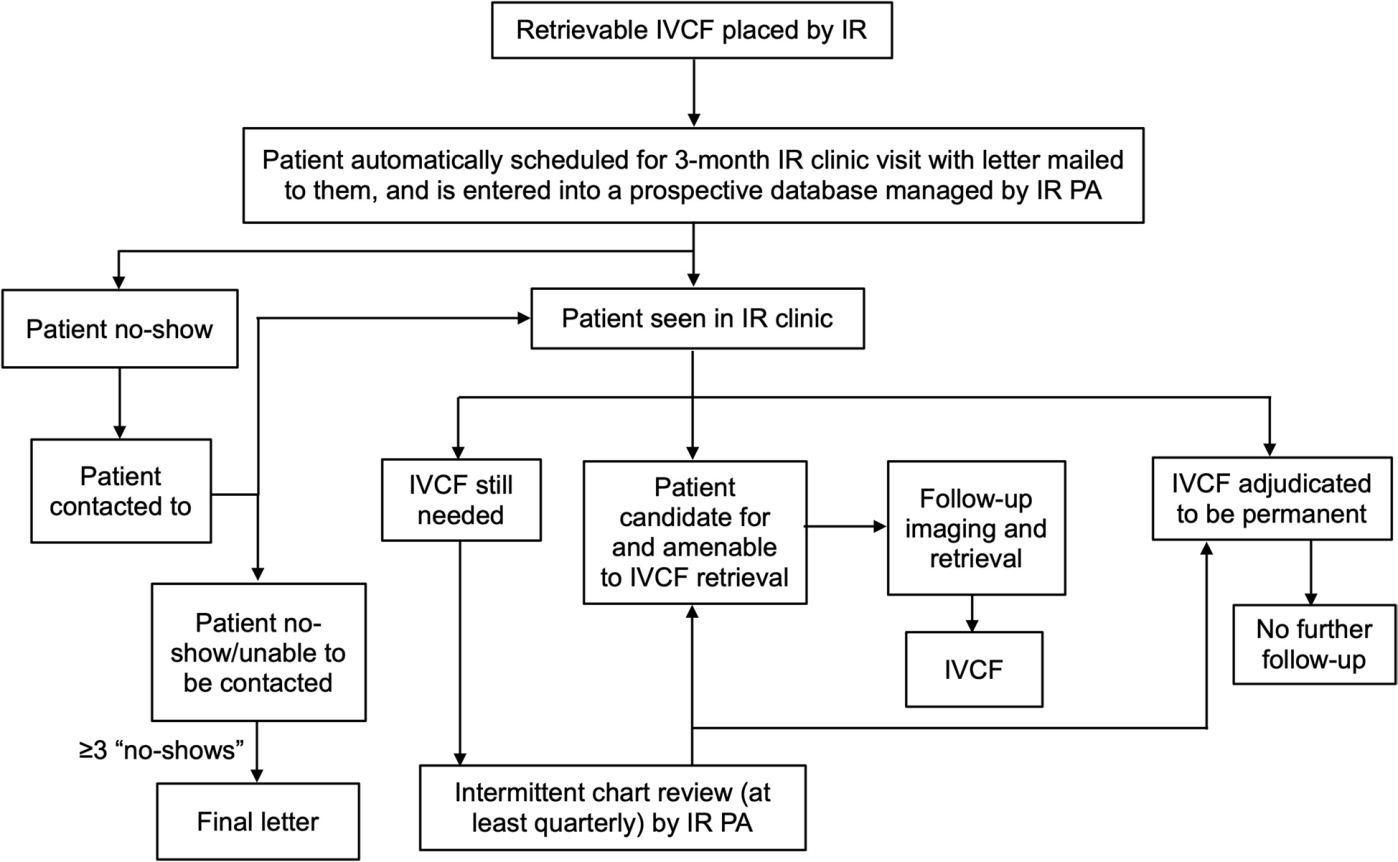
Flowchart of methodology for tracking of retrievable inferior vena cava filters (IVCF) placed by interventional radiology (IR) and management of follow-up. Patient death at any time before the filter is deemed to be permanent is recorded as such in the database

Institutional review board approval was granted (protocol #829479). The project was initiated in 2013 in an effort to more effectively follow-up patients in whom retrievable IVC filters are placed. Patients with retrievable IVC filters placed by IR at a single tertiary institution are tracked in a prospective registry, and follow-up is organized by a designated IR physician assistant (PA). No permanent IVC filters are included. The IVC filter follow-up process is as follows (Fig. 1): The patient is automatically scheduled for a follow-up clinic visit 3-months after filter placement with the physician who placed the filter. A letter is also mailed immediately with information about the filter being placed and the clinic visit date and time. These patients do not require imaging prior to the appointment. If the physician deems that filter is amenable to removal, he/she orders any necessary imaging at time of follow-up appointment on a case-by-case basis, for example if the filter has been in place for an extended period (usually at least 1 year) or there are symptoms concerning for filter-related complications, such as caval thrombosis. The PA also documents updated information, including filter status, adverse events, communications, and appointments, in a OneDrive (Microsoft Corporation, Washington, USA) spreadsheet.

If the patient does not show up for the follow-up appointment, the administrative assistant then calls the patient directly to try to reschedule and/or assess the reason for the missed appointment; all communication is documented on the spreadsheet. If the patient cannot be reached at first phone call, another letter is sent to the patient’s listed mailing address communicating the need for appointment rescheduling and asking that they contact the IR section to discuss follow-up. If they still need the filter, the IR PA intermittently reviews the electronic medical record (EMR) at least quarterly and contacts the patient by phone when filter removal is deemed appropriate. If the patient “no-shows” three times, then the administrative assistant notifies the PA and IR attending, and they make a determination to send a letter to the patient stating that several attempts have been made to evaluate the status of filter and to instruct the patient to contact IR when they are able to be evaluated.

If the filter is appropriate for removal and the patient is agreeable to proceed, a procedure date is scheduled, and the filter is removed as an outpatient IR procedure. The retrieval technique, whether via routine snare versus complex retrieval with forceps, is determined by the interventional radiologist [8]. Alternatively, if the patient requires a long-term filter, such as due to ongoing contraindication to anticoagulation or ineffectiveness of anticoagulation alone, the case is reviewed by the interventional radiologist, who could engage the patient and other clinicians, including hematology and oncology, to come to an agreement. If it is agreed upon that the filter remain in place indefinitely, the filter is adjudicated to be “permanent,” and no follow-up or retrieval is scheduled. In addition, patients who die with a filter in place are documented in the spreadsheet. Thus, filter outcomes are defined as one of the following categories: (a) retrieved, (b) permanent, (c) retrievable and either being monitored for need for filtration or is being scheduled for follow-up, (d) died with filter in place, and (e) unable to be contacted/lost to follow-up. Patients remain in the database for contact if they fall under (c). Data from medical record review were updated in October 2024. Filters placed outside of IR, such as by vascular surgery, are not included in the database.

Retrospective review of removal rates of all retrievable IVC filters was performed. Patient data characteristics, including age and sex, were also collected. Data are reported as frequencies and performed using StataSE 14.2 (StataCorp, College Station, TX).

## Results

**Fig. 2:**
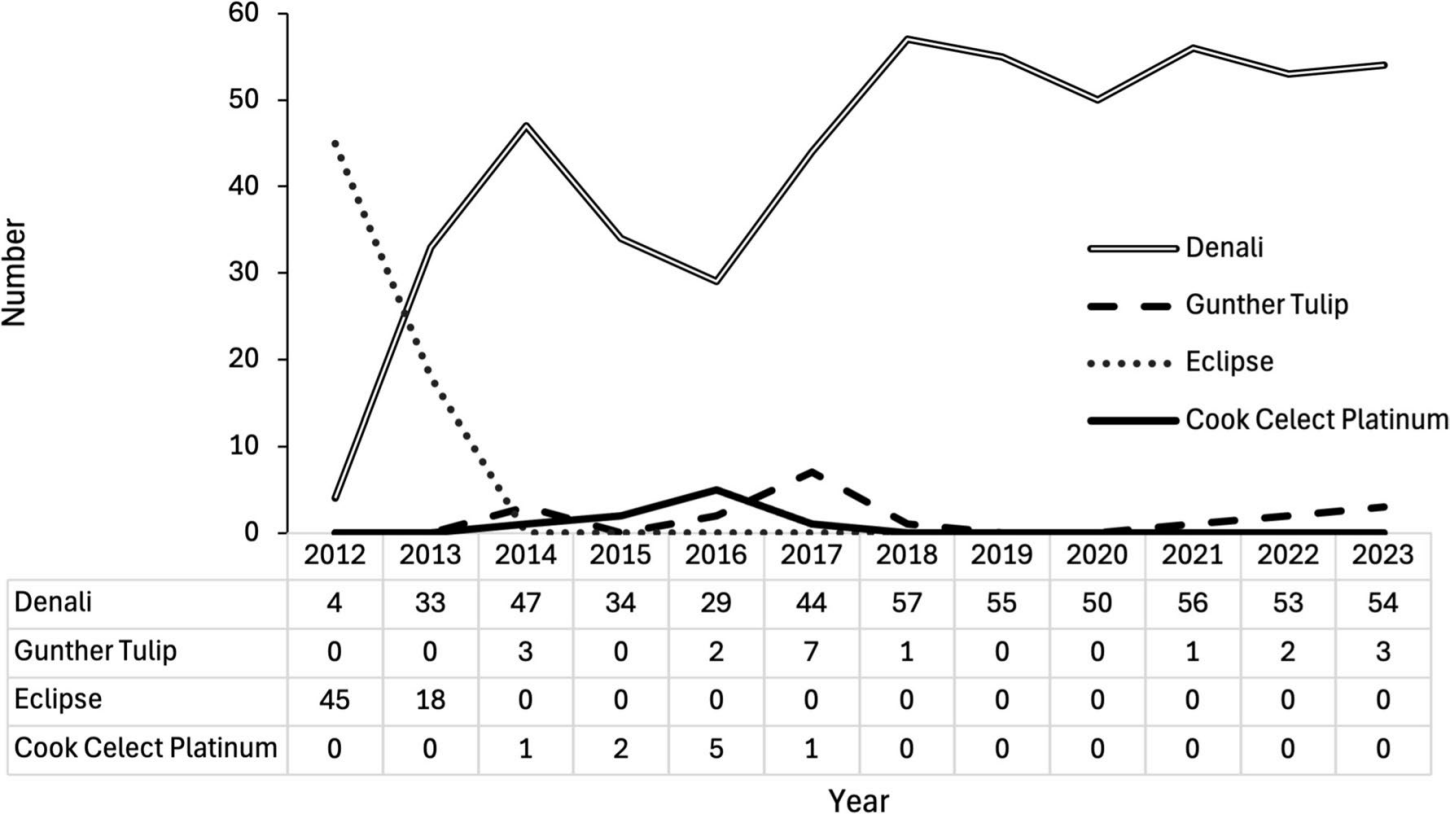
Longitudinal trend of retrievable inferior vena cava filter placements by filter type, 2012–2023

From 2012 to 2023, a total of 607 retrievable IVC filters were placed by IR (Table 1): 516 Denali (Bard), 63 Eclipse (Bard), 19 Günther Tulip (Cook Medical), and 9 Celect Platinum (Cook Medical) (Fig. 2). Table 2 lists characteristics of patients in whom filters were placed, including 53.7% males with a median age of 63.2 years at time of filter placement. The most common indications for filter placement were a contraindication to anticoagulation in patients with acute venous thromboembolism (72.8%) and pre-operative placement in patients with venous thromboembolism (19.6%).

**Table 1:** Characteristics and outcomes of retrievable IVC filter placements and removals, 2012–2023

Year	Category, N (%)				
	**Total filters placed**	**Filters retrieved**	**Filters deemed permanent**	**Died with filter**	**Alive with non-permanent filter**
**2012**	49 (8.1)	22 (8.5)	6 (8.0)	19 (7.5)	2 (10.5)
**2013**	51 (8.4)	26 (10.0)	8 (10.7)	17 (6.7)	0 (0.0)
**2014**	51 (8.4)	18 (6.9)	5 (6.7)	26 (10.3)	2 (10.5)
**2015**	36 (5.9)	12 (4.6)	7 (9.3)	16 (6.3)	1 (5.3)
**2016**	36 (5.9)	18 (6.9)	4 (5.3)	14 (5.5)	0 (0.0)
**2017**	52 (8.6)	19 (7.3)	5 (6.7)	26 (10.3)	2 (10.5)
**2018**	58 (9.6)	21 (8.1)	12 (16.0)	24 (9.5)	1 (5.3)
**2019**	55 (9.1)	18 (6.9)	2 (2.7)	33 (13.0)	2 (10.5)
**2020**	50 (8.2)	23 (8.8)	7 (9.3)	20 (7.9)	0 (0.0)
**2021**	57 (9.4)	31 (11.9)	4 (5.3)	22 (8.7)	0 (0.0)
**2022**	55 (9.1)	28 (10.8)	10 (13.3)	16 (6.3)	1 (5.3)
**2023**	57 (9.4)	24 (9.2)	5 (6.7)	20 (7.9)	8 (42.1)
**Total**	**607**	**260**	**75**	**253**	**19**

**Table 2:** Characteristics of patients with retrieval IVC filters placed, 2012–2023

Category	Subgroup	Count
Sex (N (%))	Male	326 (53.7)
	Female	281 (46.3)
Age at filter placement (*N* = 607)	Mean ± SD	61.7 ± 13.9
	Median	63.2
	Range	18.8–95.0
Age at filter removal (*N* = 260)	Mean ± SD	59.1 ± 14.4
	Median	61.3
	Range	21.1–90.2
Indication for IVC filter placement (N (%))	Contraindication to anticoagulation with DVT/PE^a^	442 (92.4)
	Prophylaxis in high-risk trauma	14 (2.3)
	Failure of anticoagulation in patient with DVT/PE	12 (2.0)
	Limited cardiopulmonary reserve and DVT/PE	11 (1.8)
	Progression of DVT/PE despite anticoagulation	5 (0.8)
	High fall risk with DVT/PE	3 (0.5)
	Poor anticoagulation compliance with DVT/PE	1 (0.2)

^a^Includes complication of anticoagulation necessitating cessation (e.g. bleeding) and conditions precluding anticoagulation (e.g., recent bleeding, intracranial abnormality, peri-operative setting, thrombocytopenia)

*DVT/PE* deep vein thrombosis/pulmonary embolism, *IVC* inferior vena cava, *SD* standard deviation

**Fig. 3:**
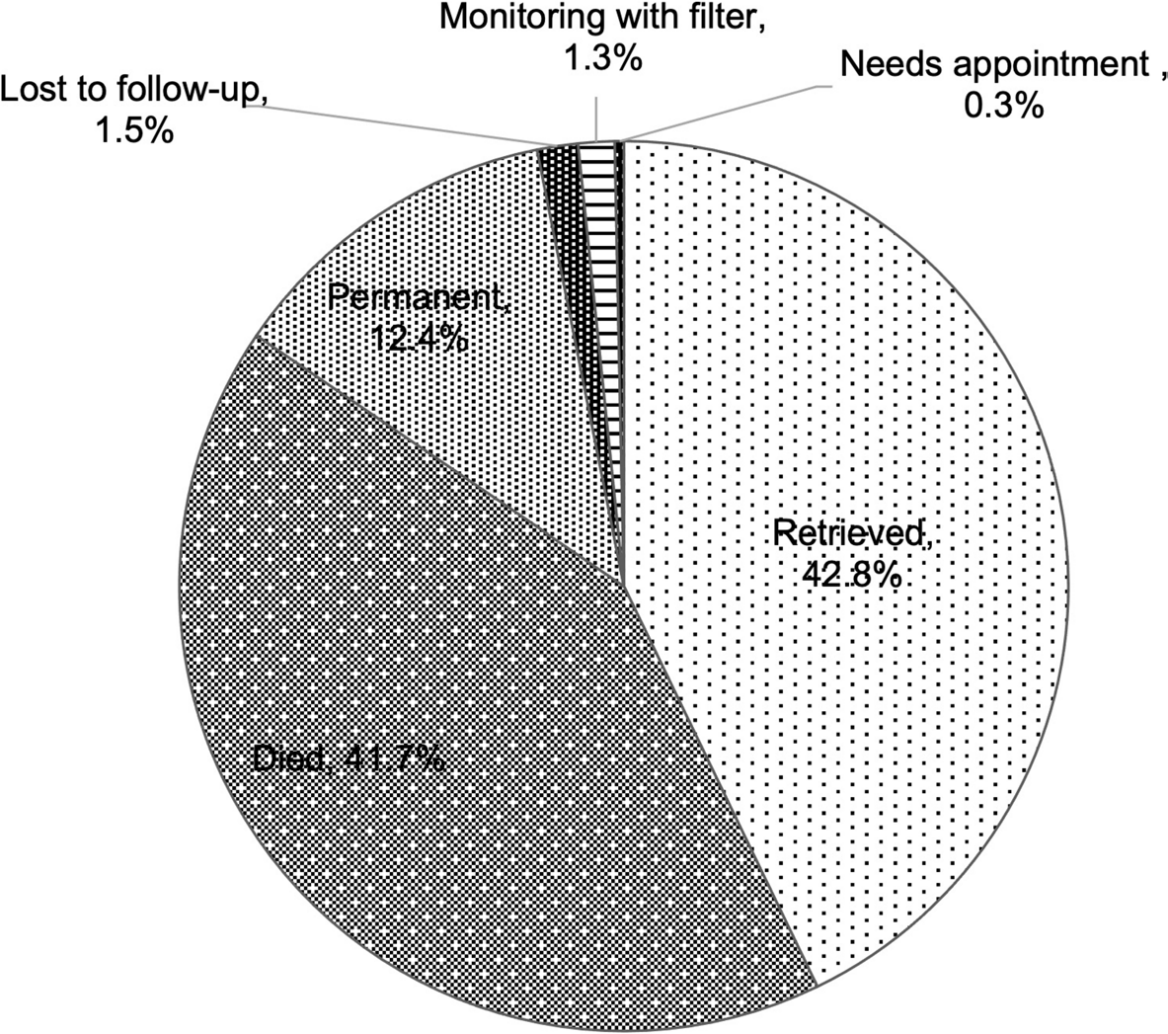
Outcome of patients with a retrievable inferior vena cava filter, including 3.1% (*N* = 19) that had not yet been retrieved or been deemed permanent

Of the filters placed, 42.8% (260/607) were retrieved (97.7% by IR at this institution, 2.3% either at an outside institution or not by IR), 12.4% (75/607) were deemed to be permanent, and 41.7% (253/607) died with the filter in place, none of which the primary cause of death was attributed to the filter (Fig. 3, Table 1). Of the 254 patients with attempted filter retrieval by IR at this institution, all were removed (100% technical success rate), and 19 (7.5% of filters retrieved, 3.1% of filters placed) were complex retrievals with endobronchial forceps [9]. Excluding patients either with a filter deemed permanent or who died before retrieval, 93.2% (260/279) of filters were retrieved during the study period. Mean (± standard deviation) and median dwell times of retrieved filters were 221 (± 303) and 133 days, respectively, with a range of 5 to 2673 days.

The remaining 3.1% (19/279) comprised those alive with a filter that had not yet been retrieved and had not been deemed permanent. Of this cohort, 8 (42.1%) still needed the filter and were being monitored to determine timing of follow-up from the IR PA, including 7 patients with filters placed in 2023; 2 (10.5%) needed an IR appointment; and 9 (47.4%) were lost to follow-up, having been sent a final letter of communication due to repeated no-shows and/or inability to reach the patient via phone or mail despite multiple attempts (Fig. 3). If patients who were being monitored or needed an appointment get their filter retrieved, the potential maximum retrieval rate would be 96.8% (270/279). Overall, 98.5% (598/607) of filters placed between 2012 and 2023 were appropriately accounted for.

## Discussion

Over the past 15 years, there has been rising awareness about the need for patient follow-up and removal of retrievable filters when no longer needed, especially due to higher adverse events with longer dwell times [3, 5, 10]. These risks include IVC thrombosis, filter migration and fracture, and symptomatic strut penetration [5, 10]. The cited risks of filter retrieval vary although are generally reported to be < 10%, with higher rates of complications associated with longer filter dwell times and need for advanced retrieval techniques [11]. However, retrieval rates have continued to be suboptimal due to a variety of reasons, with loss of patient follow-up cited as the primary cause [5, 12–14]. This study provides an example of a feasible and successful long-term tracking program for retrievable IVC filters.

The program is managed prospectively by an IR PA, in close consultation with the institution’s interventional radiologists. No proprietary tracking software or commercial database is needed, and the rates of filter accountability/follow-up are higher than reported by multiple other interventions [6, 7, 13, 15–20]. Furthermore, an established IR PA and clinic coordinator take on the responsibilities of maintaining the registry and contacting patients without needing to hire new personnel. While a PA is not a mandatory component of a filter tracking program depending on personnel availability at other institutions and IR practices, they can help facilitate centralization and organization across the system as a single point of contact. Patients with retrievable filters are also evaluated in the preexisting IR clinic structure without needing to create a dedicated IVC filter clinic. Importantly, the EMR is leveraged to automate scheduling and follow-up reminders to minimize the manual workload. While no agreed-upon definition of filter retrieval rate exists, the calculation of eligible patients in this study has been used in multiple previous studies; in addition, the 93.2% retrieval rate among eligible patients is comparable to that reported by Sheehan et al. (92.5%), Minocha et al. (91% in post-clinic years when excluding filters deemed “permanent”), and Lee et al. (92%) [17, 21, 22]. This initiative highlights the importance of continuous and active monitoring of retrievable IVC filters in a systematic manner driven by responsibility placed on the clinicians who placed the filter, as also advocated by multiple studies and guidelines [3, 7, 23].

Several limitations were present in this study. Comparative data were not available for statistical analysis, as this was performed at a single institution, and while filters placed by IR were tracked, the process was not standardized until 2012. However, qualitative comparison with companion studies about filter retrieval rates and initiatives to improve filter retrieval can be made. In addition, filters placed outside of IR at this institution, including vascular surgery, were not captured in the database. Short- and long-term adverse event data were not recorded in this database and were not specifically the focus of this study, similar to prior studies on filter retrieval initiatives as documented by Goodin et al. [6]. The percentage of patients who died with a filter in place is comparable to other studies evaluating long-term outcomes of patients with IVC filters [24, 25]. Furthermore, this initiative was coordinated to a single academic center so cannot be universally extrapolated depending on practice environment but could be adapted to individual institutions to improve filter retrieval rates on a larger scale. Analyses on the long-term impact on filter complications and cost effectiveness were not performed and are an opportunity for future investigations, although long-term adverse effects associated with indwelling filters have been well described [3, 26].

## Conclusions

This study provides details of a feasible and effective filter management program utilizing a prospectively maintained database, with 1.5% of patients lost to follow-up and an adjusted filter retrieval rate of 93% (potentially up to 97%) at the study endpoint. This initiative can serve as a framework for justifying and creating a structured IVC filter follow-up program to improve filter accountability on a broader scale.

## Data Availability

The datasets used and/or analysed during the current study are available from the corresponding author on reasonable request.

## Abbreviations

EMR: Electronic medical record
FDA: Food and Drug Administration
PA: Physician assistant
IVC: Inferior vena cava
IR: Interventional radiology
